# Health Tract, No. 107

**Published:** 1862-10

**Authors:** 


### HEALTH TRACT, No. 107.

### SMALL-POX.

It should be distinctly kept before the minds of the people that vaccination is an almost perfect preventive of small-pox until the age of puberty, (say fifteen,) but that after that time it becomes less and less efficacious until twenty-five, when the system becomes less susceptible to the disease up to thirty-five, when the predisposition to small-pox seems to die out altogether. The specific inference is, that every child ought to be re-vaccinated on entering the fifteenth year.

To show the preventive power of vaccination, statistics prove that before vaccination, or even inoculation, was practiced or known in Boston, to wit, 1721, (the year of its first trial in England by Lady Mary Wortley Montague on her own daughter,) one half of the entire population lay sick of the disease at the same time, and one out of every twenty-seven died of it—which, at the same rate, would kill over thirty thousand persons in New-York City alone—while the total deaths from all causes in a single year was less than twenty-three thousand. In 1792, forty-six per cent—forty-six persons out of every hundred, in Boston, had small-pox at the same time. But a few years later, when vaccination was generally practiced, many city physicians did not see a single case of small-pox in a period of twenty months, and during a period of twenty-eight years less than three persons a year died of small-pox in Boston. During that time the law was, that any person having small-pox should be at once conveyed to a house for that purpose, removed from all other habitations. But in 1838 that law was repealed. From this cause, together with a growing inattention to vaccination, the influx of foreigners, and the more crowded conditions of dwellings, small-pox is becoming more common; and ninety persons died of small-pox the very year after the above law was repealed, and three hundred and eighteen died of it during the twenty-one months ending October, 1860. There ought to be a law compelling vaccination within a year after a child's birth, and a re-vaccination on entering the fifteenth year. One of the greatest difficulties in the way of securing vaccination and re-vaccination, is the want of facilities for getting pure vaccine-matter from a perfectly healthful subject—there seeming to be a very general impression, groundless as is supposed, that the vaccine-matter from a diseased subject will communicate that disease to the person vaccinated, or will in some way have a deleterious effect on the constitution. Some water-cure journals, which are generally read by ignorant and uninformed persons, these unfortunately being the large majority in all communities, have disseminated the doctrine that vaccination is rapidly scrofulizing the civilized world—one of those dogmatic, impudent assertions, which a reckless ignoramus is very capable of making. Reckless enough to say any thing, without having sense enough to prove any thing. It is merely offered as a suggestion, that the Legislature should appoint an educated, experienced, and respectable physician in each county-town and in each ward of every city, whose duty it should be to keep a pure matter always on hand, and who should be paid by the State for every successful vaccination or re-vaccination made. The subject certainly merits the prompt attention of the authorities.

